# Fabrication of ZnO Ceramics with Defects by Spark Plasma Sintering Method and Investigations of Their Photoelectrochemical Properties

**DOI:** 10.3390/nano11102506

**Published:** 2021-09-26

**Authors:** Liren Zheng, Mu Liu, Haipeng Zhang, Zhaoke Zheng, Zeyan Wang, Hefeng Cheng, Peng Wang, Yuanyuan Liu, Baibiao Huang

**Affiliations:** 1 State Key Laboratory of Crystal Materials, Shandong University, Jinan 250100, China; 201720279@mail.sdu.edu.cn (L.Z.); liumu@mail.sdu.edu.cn (M.L.); 201820321@mail.sdu.edu.cn (H.Z.); zkzheng@sdu.edu.cn (Z.Z.); chenghefeng@sdu.edu.cn (H.C.); pengwangicm@sdu.edu.cn (P.W.); yyliu@sdu.edu.cn (Y.L.); 2 School of Physics and Electronic Engineering, Taishan University, Tai’an 271000, China

**Keywords:** ZnO ceramic, spark plasma sintering, oxygen vacancies, interstitial zinc, electrical properties, photoelectrochemical properties

## Abstract

ZnO, as an important semiconductor material, has attracted much attention due to its excellent physical properties, which can be widely used in many fields. Notably, the defects concentration and type greatly affect the intrinsic properties of ZnO. Thus, controllable adjustment of ZnO defects is particularly important for studying its photoelectric properties. In this work, we fabricated ZnO ceramics (ZnO(C)) with different defects through spark plasma sintering (SPS) process by varying sintering temperature and using reduction environment. The experimental results indicate that the changes of color and light absorption in as-prepared ZnO originate from the different kinds of defects, i.e., oxygen vacancies (V_O_), interstitial zinc (Zn_i_), and Zinc vacancies (V_Zn_). Moreover, with the increase in calcination temperature, the concentration of oxygen defects and interstitial zinc defects in the ceramics increases gradually, and the conductivity of the ceramics is also improved. However, too many defects are harmful to the photoelectrochemical properties of the ceramics, and the appropriate oxygen defects can improve the utilization of visible light.

## 1. Introduction

Zinc oxide (ZnO) has been paid much attention due to its wide bandgap (3.37 eV), the large exciton binding energy (60 mV), high transparency, non-toxicity, bio-compatibility, and low cost. It has been extensively studied and widely used in many fields, such as optoelectronic devices [1], piezoelectric transducers [2], and gas sensors [3], etc. Similar to the other semiconductor materials, the intrinsic properties of ZnO are closely dependent with the defects within it. Thus, tuning the defects of ZnO could subsequently lead to some new applications. For example, the change in defect concentration affects the electrical properties of boundary properties in ZnO piezoelectric ceramics [4], and oxygen vacancy (V_O_) is mainly responsible for the n-type conductivity in ZnO [5]. Oxygen defects are excellent because of their dilute magnetism, catalytic ability enhancement, adsorption and activation of carbon dioxide, etc. [6,7,8]. Zinc vacancy (V_Zn_) is the acceptor of point defects, and its formation process is restricted by experimental conditions [9]. As a shallow donor in ZnO, interstitial zinc (Zn_i_) can effectively increase its conductivity and carrier concentration [10,11].

To date, various strategies have been developed for the defect engineering of ZnO, such as ball milling [12], decomposition of ZnO_2_ [13], hydrogen reduction [14], etc. Furthermore, various ZnO containing different kinds of defects have been fabricated. Lu et al. fabricated black H-ZnO nanostructures by hydrogenating the ZnO film on the FTO substrate [14]. Owing to the presence of oxygen vacancies (V_O_) and interstitial hydrogen (H_i_) in H-ZnO, it exhibits strong light absorption in the range of 300–700 nm. Red ZnO containing interstitial zinc (Zn_i_) defects has also been reported by Hofmann et al. [15], which was fabricated by annealing ZnO single crystal in presence of Zn vapor atmosphere. Moreover, recently, we have fabricated yellow ZnO containing V_O_ defects by direct thermal decomposition of ZnO_2_, which extend the light absorption edge of ZnO from 380 nm to 480 nm [13]. Although various ZnO containing different kinds of defects have been reported, the type of defects in ZnO is usually formed under different synthetic conditions, and the concentration of the defects in the as-prepared ZnO is usually low, which greatly limits the research on the intrinsic properties of ZnO. If the defects in ZnO could be tuned in larger range, the properties of ZnO could be further optimized.

The sintering methods for preparing zinc oxide ceramics include cold sintering [16,17,18], ultra-high temperature sintering [19], flash sintering [20], and spark plasma sintering [21,22]. Spark plasma sintering (SPS) is a rapid sintering technique, which is widely used to fabricate metals [23,24], metallic alloys [25,26], carbide [27], various MAX-phase materials [28], and metallic oxide (ZnO, AZO) [29,30], etc. Different from the conventional sintering method, SPS can fabricate ceramics in very short time under low voltage and high direct current condition. Additionally, SPS can also provide various sintering environments, such as vacuum atmosphere, oxygen-deficient environment, pulse current, high temperature, and high mechanical pressur, which could be an ideal method to fabricate ZnO with tunable defects.

In this work, we fabricated ZnO ceramics (ZnO(C)) with different kinds of defects, namely, oxygen vacancies (V_O_), zinc vacancies (V_Zn_), and interstitial zinc (Zn_i_), through SPS method. The defect types and concentrations of the as-prepared ZnO have been extensively investigated. The oxygen defects and the electron mobility increase with the increase in sintering temperature. Meanwhile, the light absorption edge of the ZnO shift from 384 nm to 600 nm continuously. Notably, too many defects will greatly reduce the photoelectric properties of ZnO ceramics. However, the existence of appropriate concentration of oxygen defects in ZnO is beneficial to the utilization of visible light.

## 2. Experimental Procedures

### 2.1. Preparation of ZnO Ceramics Using SPS Method

The raw material is pure zinc oxide powder directly purchased from Sinopharm Chemical Reagent Co., Ltd (Shanghai, China) without further treatment. Before the experiment, zinc oxide powder was put in an oven at 500 °C for 2 h to remove organic matter and water. The particle size of ZnO powder was 50–500 nm, and the specific surface area was 13.2 m^2^/g. The preparation process of ceramics is as follows. First, 70 mmol of zinc oxide powders was put into a graphite mold, and then the mold was put into SPS furnace (Mini-SPS, Shang Hai Chen Hua Technology Co., Ltd, Shanghai, China). Then, the vacuum pump turned on to discharge the air in the furnace. When the vacuum value was about 10 Pa, the furnace was heated to 600 °C at 120 °C/min and kept for 20 min at the temperature. Then, the sintering temperature was raised to the target temperature (700, 900 or 1100 °C) at 80 °C/min and kept at this temperature for 30 min. The pressure was 66 MPa on the ceramic during the sintering process. Finally, when the temperature dropped to room temperature, the ceramics were taken out from the graphite dies and the graphite was removed on its surfaces with sandpaper. Then, the density of the ceramics was measured using Archimedes’ principles. The ceramic was cut into thin slices (sizes: 5.0 × 5.0 × 0.5 mm) with a diamond wire cutting machine. For the sake of simplicity, the ceramics prepared at 700, 900, and 1100 °C were labeled with ZnO(C)-1, ZnO(C)-2, and ZnO(C)-3, respectively.

When the sintering temperature was 700 °C, the volume of ZnO powder obviously decreased, and the grain size increased with pore exclusion. When the sintering temperature was lower than this temperature, ceramics were easy to break. A small amount of zinc oxide was decomposed at the 900 °C vacuum atmosphere to form a small amount of interstice zinc in ceramics, and the density of ceramics was further increased at the sintering temperature. At a higher sintering temperature, the decomposition of zinc oxide ceramics was expected to intensify. At 1100 °C, the gap zinc formed in ceramics reached a higher value. When the sintering temperature continued to increase, the decomposition of zinc oxide was expected to be very serious. So, we chose these three representative sintering temperatures to prepare zinc oxide ceramic.; The holding time of sintering ceramics was 30 min, in order to make the formation of uniform defect distribution in the ceramics.

### 2.2. Preparation of ZnO Ceramics Using Traditional Sintering Method

Next, 2 g of ZnO powder was put into a stainless-steel mold with a diameter of 15 mm for forming green body. Then, the disc was put into a rubber bag, following by removing the air, and the rubber bag was put into a cold isostatic press (Shenyang Kejing Auto-Instrument Co., Ltd., Shenyang, China) to form a ceramic body under 250 MPa pressure for 8 min. Then, the ceramic body was put into a atmosphere tubular furnace and sintered in Ar gas at 700, 900, and 1100 °C for 30 min, respectively. After the furnace temperature dropped to room temperature, the three samples were taken out from the furnace, and marked with ZnO(C)^1^, ZnO(C)^2^ and ZnO(C)^3^, respectively.

### 2.3. Heat Treatment Process of Ceramics in Air and Vacuum

To further study the spectral and color change in ceramics in different atmospheres, the ceramic samples were annealed at 500 °C in air or at 1100 °C in vacuum atmosphere.

Three slices samples were annealed at 500 °C in air atmosphere. The process is as follows. First, the ZnO(C)-1, ZnO(C)-2, and ZnO(C)-3 slices samples were put into a muffle furnace. Then, the furnace was heated to 500 °C at a heating rate of 5 °C min^−1^ and maintained at the temperature for 5 h. Those samples were taken out from the furnace when the temperature drops to room temperature. Then, we marked those ceramics treated in air with ZnO(C)-1A, ZnO(C)-2A, and ZnO(C)-3A, respectively.

In vacuum atmosphere, three samples (ZnO(C)-1B, ZnO(C)-2B and ZnO(C)-3B) were placed in a high vacuum furnace (Shanghai Chenhua Technology Co., Ltd., Shanghai, China) in a vacuum atmosphere. When the furnace temperature is 1100 °C and the atmospheric pressure in the furnace was 0.01~0.001 Pa, the three samples were annealed for 1 h. Then. the three samples were taken out after the furnace temperature dropped to ambient temperature. For simplicity, the three samples were labeled as ZnO(C)-1B, ZnO(C)-2B, and ZnO(C)-3B, respectively.

### 2.4. Preparation of Ceramic Photo Electrode

The above six ceramic slices were cut into 120–180-μm-thick sheets. Then, those sheets were treated by ultrasonic cleaning in xylene and ethanol to remove the paraffin on the surfaces of these sheets. Then, they were placed in a muffle furnace at 500 °C for 3–5 min to remove the residual organic matter on their surfaces. So, one side of the treated sheet (the opposite side contacts the electrolyte) was coated with indium–gallium (In–Ga) alloy, and the prepared copper wire was fixed on the surface of the In–Ga alloy with silver glue, and then the sheet was packaged with epoxy resin. Only the 2–6-mm^2^ area was reserved to test the photoelectric performance.

### 2.5. Characterization

The crystal phase structure was analyzed on the Bruker D8 advance powder diffractometer with Cu Kα X-ray radiation (λ = 0.15418 nm). The scanning electron microscope (SEM) photos of the ceramics were characterized by a Hitachi S-4800 microscope with an accelerating voltage of 5 kV. The X-ray photoelectron spectroscopy (XPS) measurements were performed on an ESCALAB 250 photoelectron spectrometer (employing Al Kα radiation, E = 1486.6 eV). The C 1 s line located at 284.6 eV was used as the calibration position for all element data. The diffuse reflectance spectra were measured using Shimadzu UV 2550 UV-vis spectrometer equipped with an integrating sphere in the wavelength range of 200–800 nm. The Raman spectra were obtained by excitation of the samples with a 473-nm laser on a LabRAM HR800 Raman spectrometer (Horiba Jobin Yvon, (Paris, France). The carrier concentration, mobility, and resistivity were measured using a MMR K2500 Hall effect test system. The grain size distribution of the ceramic samples was measured and calculated through the SEM images of ceramics. We used a scale to measure the grain size in the photo. After repeated measurements of irregular grains, the results were averaged to obtain the grain size.

### 2.6. Measurement of Photoelectrochemical Properties of ZnO Electrodes

The photoelectrochemical properties of six electrodes were carried out at three electrode system, in 0.1 M Na_2_SO_4_ electrolyte (pH = 6.8), in which the prepared electrode was used as the working electrode, the Pt plate was used as the counter electrode, and a saturated calomel electrode (SCE) was used as the reference electrode. Then, the measurement data were recorded by electrochemical workstation (Shang Hai ChenHua Electrochemical Analyzer/Workstation Model CHI600E, Shanghai, China). All measurements were carried out under simulated AM (Air Mass) 1.5 G solar illumination provided by a 300-W Xe arc lamp equipped with an AM 1.5 G filter ((Perfectlight, Beijing, Co., Ltd., Beijing, China), 100 mW/cm^2^). The current was represented by I_1.5_ at 1.23 V_RHE_. In this work, all the data were obtained using the saturated calomel electrode (SCE), so the bias will be converted to reversible hydrogen electrode (RHE) by using the equation:

(1) $$E_{RHE}=E_{SCE}+0.591pH+0.241$$

To study the response of ZnO(C) electrode with different defect concentration to visible light, their photocurrent density-bias curves (J vs. V) were taken at three electrodes system with a scan rate of 20 mV/S in 0.1 M Na_2_SO_4_ electrolyte (PH = 6.8) under visible light irradiation by a 300-W Xe arc lamp equipped with a UV/IR cut filter (λ ≥ 420 nm). The current is represented by I_v_ at 1.23 V_RHE_.

## 3. Results and Discussion

### 3.1. X-ray Diffraction (XRD)

**Figure 1:**
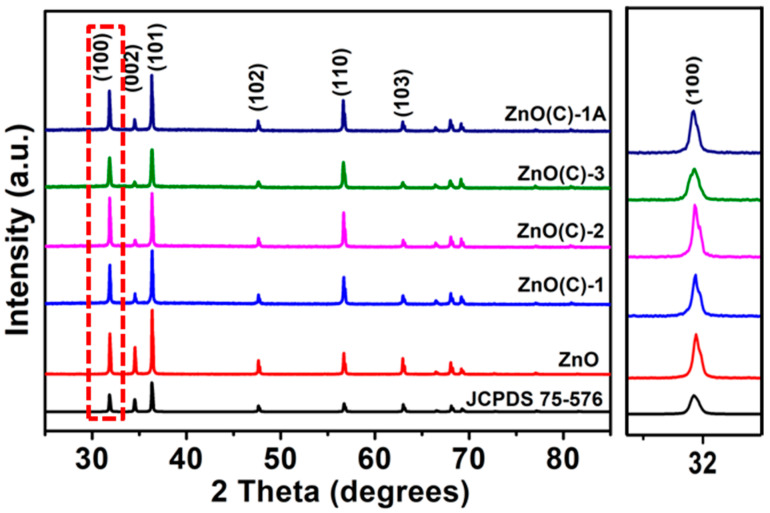
XRD patterns of ZnO powder, ZnO(C)-1 (2 and 3) and ZnO(C)-1A.

Figure 1 and Figure S1A–C show the XRD data of ZnO powder and nine ceramic samples. The six obvious diffraction peaks in ceramic samples can be observed at 2θ of 31.8°, 34.5°, 36.3°, 47.7°, 57.7°, and 63.0°, which correspond to the crystal indexes of (100), (002), (101), (102), (110), and (103), respectively. These peaks are corresponding to the standard card (No: 75-576). The ZnO(C)-1(2 and 3) samples prepared at three temperatures are hexagonal wurtzite structure, which is similar to those organized by other research groups [31]. Even if the three samples are treated in air, their structures and phase are not changed, see Figure 1 and Figure S1A. However, the diffraction intensity of all ZnO(C) samples in the [002] direction is weaker than that of ZnO polycrystalline materials prepared by other methods [17,32]. Furthermore, the intensity in [002] direction of the ceramics prepared at 1100 °C is weaker than that of the other two samples (Figure S1B). After heat treatment in air, the X-ray diffraction data of ZnO(C)-3A show that the peak intensity in the direction is still weak. There are also a number of studies showing that the electric field of the pulsed current can affect the component or defect in ceramic [21,31]. Moreover, the [002] direction in zinc oxide is the direction with the highest polarity [33]. In the process of sintering, the growth along the direction is disturbed by pulsed electric field. The XDR data of three ceramic samples treated under 1100 °C vacuum atmosphere and the ceramic samples (ZnO(C)^1^, ZnO(C)^2^, and ZnO(C)^3^) obtained by traditional sintering methods are shown in Figure S1B,C. Under the condition of high temperature (1100 °C) and vacuum atmosphere, ZnO ceramics appear decomposition phenomenon, the mass of samples after treatment is reduced than before (Table S1), and gray metallic zinc was deposited on the inner surface of quartz glass in the observation window of high temperature furnace, see Figure S1D. Although the position of the XRD peak of the ceramic samples did not change significantly, the intensity of the diffraction peak in the [100] direction decreased and widened, indicating that some defects were introduced into the ceramic during sintering [34] (Figure 1 and Figure S1A).

### 3.2. Digital Photograph and Ultraviolet-Visible Spectroscopy

**Figure 2:**
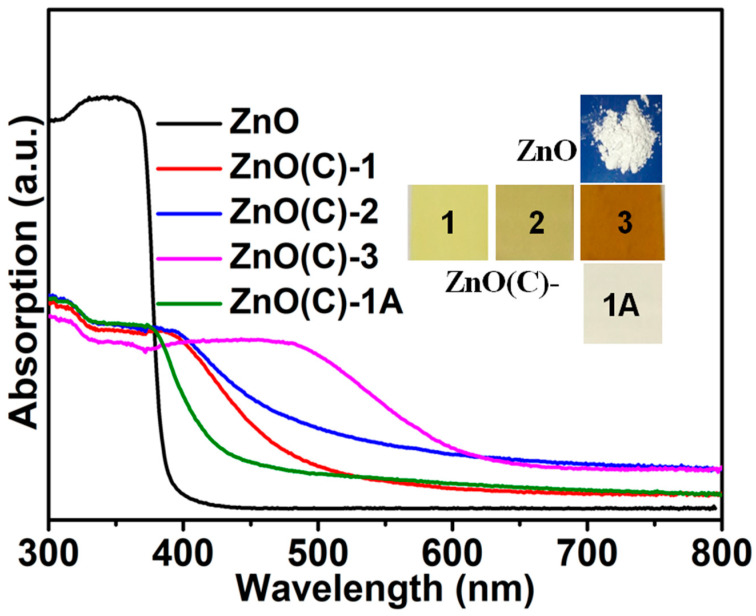
UV-Vis DRS of ZnO, ZnO(C)-1 (2 and 3) and ZnO(C)-1A samples, insert: the digital photograph of ZnO, ZnO(C)-1 (2 and 3), and ZnO(C)-1A.

The digital photograph and ultraviolet-visible spectroscopy (UV-Vis DRS) of ZnO(C)-1 (2 and 3), ZnO powder, and ZnO(C)-1A samples are displayed in Figure 2. Figure 2 shows that the color of the three ceramics (ZnO(C)-1 (2 and 3)) changes greatly at different sintering temperatures: from white to light yellow (700 °C), dark yellow (900 °C), and reddish-brown (1100 °C). Moreover, the absorption edges of the ZnO(C)-1 (2 and 3) samples shift from 384 nm (powder) to 454 nm (700 °C), 464 nm (900 °C), and 600 nm (1100 °C), respectively. After oxygen supplement, the color of the ceramic prepared at low temperature (700 °C) turns grayish-white (Figure 2), and that of ZnO(C)-2A turns pale yellow (Figure S2A). Their absorption edges shifts to 408 nm and 428 nm, respectively. However, the color of ZnO(C)-3A is still reddish brown (Figure S2A), and its absorption edge is still 600 nm. In addition, the color of the three samples treated at 1100 °C in vacuum atmosphere turned bright yellow, and their absorption edges moved to about 517 nm, see Figure S2B. The color of ZnO(C)-1, ZnO(C)-2, and ZnO(C)-3 ceramics sintered by traditional methods gradually changes from white to yellow, and their UV-Vis DRS show a similar redshift phenomenon to those prepared by SPS, but the absorption edge cannot redshift to 600 nm (Figure S2C). However, the SPS sintering method can regulate the defects and spectral properties of ZnO ceramics over a wide range (Figure 2 and Figure S2A).

The changes of color and light absorption are mainly caused by various defects (such as V_O_, V_Zn_ and Zn_i_) and defect concentration. However, the absorption edge of the two ceramics (ZnO(C)-1A and 2A) do not return to the original absorption edge at 384 nm (Figure 2 and Figure S2A), which clarifies that there are other defects (such as V_Zn_, Zn_i_) in the ZnO(C)-1 and ZnO(C)-2. The absorption edge and color of ZnO(C)-3 did not change before and after heat treatment in air. Therefore, the oxygen vacancy in ZnO(C)-3 is not the main defect, but it is the main defect in ZnO(C)-1, and the oxygen defect in ZnO(C)-2 is one of the main defects. However, in the three samples (ZnO(C)-1B (2B and 3B)) after vacuum heat treatment (Figure S2B), the content of interstitial zinc defect is greatly reduced, and the oxygen defect should be the main defect.

### 3.3. Analysis of Defect Formation

The defects in ZnO are closely related to its preparation temperature and environment. In the sintering process of ZnO ceramics, SPS equipment forms a closed, vacuum, and anoxic environment. Additionally, the preparation of the ceramics needs to be carried out in graphite mold, which is a reduction environment. At a high temperature (>900 °C), a small amount of zinc oxide that contacting with the graphite tooling is reduced to zinc by carbon, and zinc oxide is easier to decompose at high temperature and in vacuum environment provided by SPS. These are beneficial to the introduction of different defects in ZnO at different sintering temperatures. During the sintering process, the ceramic is heated by low voltage pulse direct current (DC). It will further produce defects in ZnO ceramics [31], such as reducing the zinc ions in ZnO lattice and forming zinc vacancies.

In ZnO, the formation energies of oxygen vacancy (V_O_) are lower than those of interstitial zinc (Zn_i_) and zinc vacancy (V_Zn_) [9,35,36]. Then, V_O_ is the most easily formed in the ZnO ceramics. The other two defects should be created under certain conditions, such as high temperature and high pressure. The ZnO ceramics are prepared at 700 °C (900 or 1100 °C) and under 66 MPa as well as in a vacuum atmosphere, therefore, oxygen defects first appear in ceramics. With the increase in sintering temperature, a large number of oxygen vacancies (V_O_) will be produced. Furthermore, at high temperature (≥900 °C), oxygen atoms and zinc atoms will leave the lattice point in ZnO. The oxygen atoms separated from ZnO lattice will become interstitial oxygen (O_i_) or leave ZnO ceramics. Interstitial oxygen (O_i_) has low formation energy in an oxidizing atmosphere, whereas the generation of Zn_i_ and V_O_ is favored in a reducing atmosphere [35]. Therefore, it is difficult to form interstitial oxygen (O_i_) in the ZnO ceramics. The diffusivity of Zn is much higher than that of O in ZnO [37,38,39], and ZnO has a relatively open lattice structure, with tetrahedral and octahedral sites for interstitial zinc atoms. The octahedral sites are stable positions for interstitial zinc [36,40]. Hence, Zn atoms are likely to diffuse into octahedral sites to form interstitial Zinc in our experiment. The effect of hydrogen can also be eliminated because there is no water and organic matter in the raw material. With the increase in calcination temperature, the content of oxygen vacancy (V_O_) and interstitial zinc (Zn_i_) will increase. According to the above analysis, the main defects in ZnO(C)-2 are oxygen defects and interstitial zinc, and the main defects in ZnO(C)-3 are interstitial zinc, followed by oxygen defects. Moreover, there are also a few zinc vacancies in ZnO ceramics.

To further study the oxygen vacancies, zinc vacancy and interstitial zinc in the ceramics, element states, Raman spectra, electrical property, and photoelectrochemical property of the nine samples are characterized.

### 3.4. Scanning Electron Microscopy (SEM)

The SEM images of ZnO powder, the three ceramics, and the annealed samples are revealed in Figure S3. The diameter of grain increases with the increase in sintering temperature. The grain size of ceramics increases to 400–800 nm at 700 °C, 3–6 μm at 900 °C, and 10–25 μm at 1100 °C, respectively. The SEM images show no significant changes in grain size compared with those before heat treatment in air. With the increase in grain size in ceramics, the number of grain boundaries will decrease, which is beneficial to the improvement of electrical conductivity of ceramics. At low temperature (700 °C), there are many pores in ZnO(C)-1, and its density is 98.1% of theoretical density (5.65 g/cm^3^), see Table S2. Therefore, the electrical properties of ZnO(C)-1 sample should be the worst compared with the other two samples. However, the density of the ceramics prepared at high temperatures is higher than the theoretical density (Table S2). A large amount of oxygen vacancies cause the density of ZnO ceramics to increase, and the density of the ZnO(C)-3 is 5.70 g/cm^3^.

### 3.5. X-ray Photoelectron Spectroscopy (XPS)

**Figure 3:**
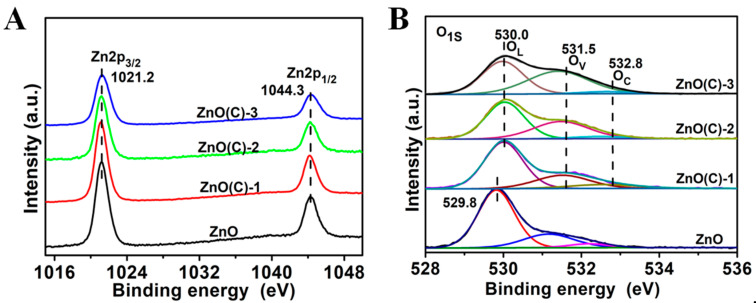
XPS patterns of the ZnO powder and ZnO(C)-1 (2 and 3), (**A**) Zn2p peak and (**B**) O1S peak.

**Figure 4:**
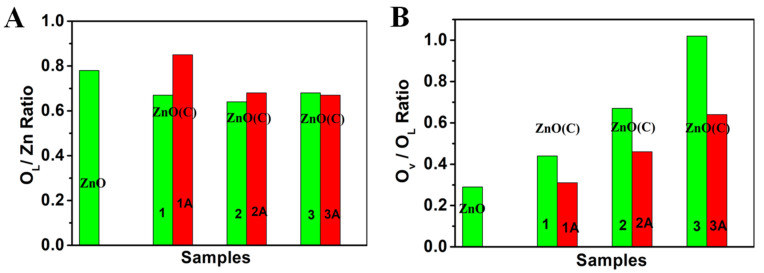
O_L_/Zn ratio and O_V_ /O_L_ ratio of ZnO, ZnO(C)-1 (2 and 3) and the ZnO(C)-1A (2A and 3A) samples, (**A**) O_L_/Zn ratio and (**B**) O_V_/O_L_ ratio.

Figure 3 and Figure S4 are the XPS of ZnO powder, ZnO(C)-1(2 and 3), ZnO(C)-1A (2A and 3A), and ZnO(C)-1B (2B and 3B) samples. The results show that the position of the Zn2p peak of the nine samples does not shift, which is 1021.20 eV and 1044.30 eV [41,42,43], respectively (Figure 3A and Figure S4A,C). The O1s peaks of the ten samples can be fitted into three peaks: 529–530 eV is the lattice oxygen peak (O_L_) [41,43,44], 530.5–531.8 eV belongs to the oxygen vacancy (O_v_) peak [43,44,45], and the 532–533 eV peak belongs to the adsorbed oxygen on the material surface [43,44]. Compared with the powder, the peak position of oxygen in ceramic samples moves slightly to the high binding energy. With the increase in temperature, the content of oxygen vacancies increases gradually in ceramics, with 0.44 and 0.67 in ZnO(C)-1 and ZnO(C)-2, respectively (Figure 3B and Figure 4B). In ZnO(C)-3, the ratio is about 1, while that of powders is only 0.33. A large number of oxygen vacancies are generated in ceramics at 1100 °C. Furthermore, the ratio of lattice oxygen (O_L_) to zinc content is the largest in powder, and decreases slightly in ceramics (see, Figure 4A). As a result, the density of ZnO ceramics increases (Table S2). However, the atomic ratio of oxygen to zinc in ZnO(C)-3 is slightly higher than that in ZnO(C)-2. This is because the zinc element in ZnO will volatilize at 1100 °C.

After calcination in air, the O1s peak in ZnO(C)-1A moved to 529.8 eV due to the decrease in oxygen vacancy (Figure S4B), which is consistent with the blue shift of the absorption edge and its grayish white change, and the content of oxygen vacancies in ZnO(C)-1A (2A and 3A) samples also decreased obviously, see Figure 4B. The data explain that the ratios of O_V_/O_L_ in the three ceramic samples decreases, which is 0.31 (ZnO(C)-1A), 0.46 (ZnO(C)-2A), and 0.64 (ZnO(C)-1A), respectively. Furthermore, the ratio of the ZnO(C)-1A is the same as that of ZnO powder. The results are consistent with the data of XPS, see Figure 3B. After calcination in vacuum atmosphere, the concentration of oxygen defects in the three samples (ZnO(C)-1B, ZnO(C)-2B, and ZnO(C)-3B) increased sharply (Figure S4D). The ratios of O_V_/O_L_ in the three ceramic samples is 1.75 (ZnO(C)-1B), 1.78 (ZnO(C)-2B), and 2.07 (ZnO(C)-3B), respectively. This fully indicates that the concentration of oxygen vacancy is very high in the ceramic treated at high temperature under vacuum condition. Those results display that the SPS method, high-temperature air atmosphere, and high-temperature vacuum condition can control the oxygen defect concentration in a large range, thus changing the optical absorption, band gap and photoelectric properties of ZnO.

### 3.6. Raman Spectroscopy

**Figure 5:**
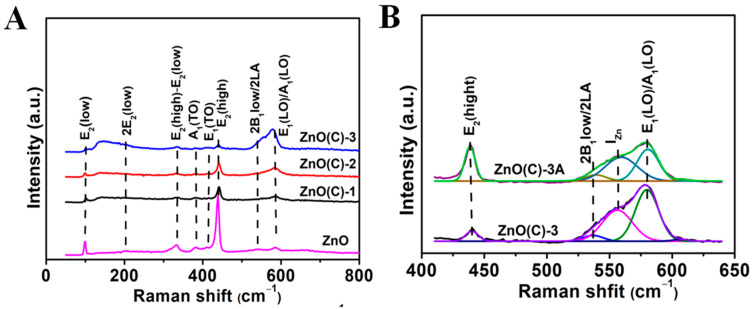
(**A**) Raman spectrum of ZnO, ZnO(C)-1 (2 and 3) ceramics and (**B**) Raman Gauss fitting curve of the ZnO(C)-3(3A) in Figure 5A and Figure S5.

Raman scattering spectroscopy was employed to identify oxygen vacancy (V_O_), zinc vacancy (V_Zn_), and interstitial zinc (Zn_i_) [33,35,46]. Figure 5 illustrates the Raman spectra of the ZnO powder and all ceramics recorded at room temperature using excitation laser 473 nm. The two most intensive modes in the Raman spectrum of ZnO powder and ceramics are E_2_ (low) at ~99 cm^−1^ and E_2_ (high) at ~439 cm^−1^, which belong to the vibrations of the zinc sub lattice and the oxygen vibration in ZnO, respectively [47,48,49,50]. The four peaks of the ZnO ceramics are 202, 330, 378, and 407 cm^−1^, corresponding to 2E_2_ (low), E_2_ (high)—E_2_ (low), A_1_(TO) (TO, Transverse Optics), and E_1_(TO), respectively.

Compared with ZnO powder, the Raman peak of ceramics moves to a higher wavenumber and the position of Raman peak of ceramic is very consistent with that of ZnO crystal or high-quality ZnO films [50,51,52], because the crystallinity of ceramics is better than that of powders [53]. There are two broad asymmetric peaks at 80~250 cm^−1^, and 500~620 cm^−1^ in the three ceramics (ZnO(C)-1 (2 and 3)), and their relative strength gradually increases with the increase in calcination temperature (Figure 5A). Especially at 1100 °C, the two peaks are strongest in the ZnO(C)-3. The 80~250 cm^−1^ broad peak (the thick light blue dotted line in Figure 5A) was due to the effect of interstitial zinc (Zn_i_) [54,55]. However, the Raman spectrum of ceramics obtained at 700 °C has this peak, which indicates that interstitial zinc (Zn_i_) appear in ZnO(C)-1 for the DC pulse current [31], but the concentration of interstitial defects is very low, not the main defects. Those show that, with the increase in sintering temperature, the Zn_i_ content in the ceramics increases, especially in the sample (ZnO(C)-3) prepared at 1100 °C, which is the main defect in the sample. The other broad peak is the overlap of three Raman modes: ~540, 560, and 588 cm^−1^, see the Gauss fitting curve of the three Raman data, see Figure 5B. The first one is generally assigned to second-order Raman mode [2B_1_low; 2LA] [56]. Previous literature reports have suggested that the 560 cm^−1^ mode is attributed to Zn type of defects [57], whereas the last two modes have been assigned as A_1_(LO) and E_1_(LO), respectively. These latter two modes are so closely spaced that their individual contributions are very much difficult. The two modes (560 cm^−1^ and 588 cm^−1^) belong to the Zn defects (I_Zn_) and oxygen disorder in ZnO lattice [10,49,58]. According to our experimental conditions and experimental process, the peak at 560 cm^−1^ should belong to the defect vibration peak of zinc vacancy (V_Zn_). After oxygen supplement in air, the intensity of peaks at 80~250 cm^−1^ and 500~620 cm^−1^ in ZnO(C)-1A (2A) samples decreased greatly, and the intensity of 437 cm^−1^ peak was enhanced, and the peak intensities at this point in ZnO(C)-3A are also greatly enhanced (Figure 5), which is consistent with the results of XPS data analysis of the three samples, see Figure 3 and Figure 4. In a high-temperature vacuum environment, zinc and oxygen elements in ZnO will break away from the lattice, then zinc oxide will decompose, and the defect of interstitial zinc will also volatilize under this condition. Therefore, after heat treatment, the defect concentration of Zn_i_ in the three samples (ZnO(C)-1B (2B and 3B)) is greatly reduced, which is confirmed by their Raman spectra. Moreover, the peak in the 500–600 cm^−1^ in Figure S5B shows that zinc defects (V_Zn_) increased in the three samples. These phenomena and results are consistent with the XRD data and the defect formation mechanism. Furthermore, the Raman spectra of the three samples (ZnO(C)^1^, (ZnO(C)^2^, and ZnO(C)^3^)) show little change. Only the two peaks related to zinc and oxygen at 99 cm^−1^ and 439 cm^−1^ were significantly weakened at high temperature (≥900 °C), see Figure S5C. Obviously, the oxygen deficiency increased, and there was a small amount of intercalated zinc in the three samples. These data correspond to their UV-Vis DRS and XRD data.

### 3.7. Electrical Property

Table S3 shows the mobility and carrier concentration of the three ceramic samples (ZnO(C)-1(2 and 3)). The results show that the electron mobility and carrier concentration increase with the increase in sintering temperature. They change from 7.94 cm^2^/vs. and 6.65 × 10^14^ cm^−3^ (ZnO(C)-1) to 26.63 cm^2^/vs. and 1.63 × 10^18^ cm^−3^ (ZnO(C)-2), and then to 38.94 cm^2^/vs. and 2.86 × 10^18^ cm^−3^ (ZnO(C)-3). The resistivity of ceramics sintered at 700 °C is the highest in the three samples (Table S3), which is mainly caused by more grain boundaries and little defects. Zinc oxide is an n-type semiconductor, and its carrier concentration is generally between 10^16^ and 10^17^ cm^−3^ [44]. The high sintering temperature leads to the increase in defect concentration, especially the oxygen vacancies (V_O_), interstitial zinc (Zn_i_) defects, and grain size. This then led to the decrease in resistivity and the increase in carrier concentration [10,11].

### 3.8. Photoelectrochemical Property

**Figure 6:**
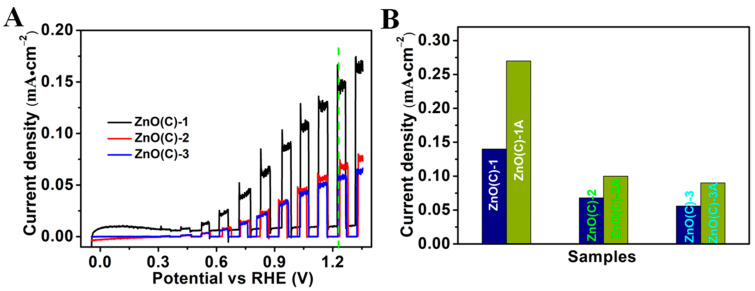
Photocurrent density versus applied potential (J-V) curves measured for the ZnO(C)-1 (2 and 3) photoanodes with a scan rate of 20 mV/s under AM 1.5 G illumination in 0.1 M NaSO_4_ solution (PH = 6.8), (**A**) ZnO(C)-1 (2 and 3) and (**B**) photocurrent values of the six photoanodes at 1.23 V_RHE_.

To further explore the influence of these defects on ZnO(C) samples, we tested the photoelectric properties of six photoanodes. These results are shown in Figure 6 and Figure S6. Figure 6A illustrates that the photocurrent of the ZnO(C) electrodes decreases with the increase in defect concentration and defect types. The current value of the ZnO(C)-1 is 0.14 mA/cm^2^ at 1.23 V_RHE_, which is better than that of the other samples. Furthermore, the current of their values is 0.068 mA/cm^2^ (900 °C) and 0.056 mA/cm^2^ (1100 °C), respectively, see Figure 6. After calcination in air, their photoelectric properties are improved (Figure S6). The photocurrent of the ZnO(C)-1A sample with the lowest defect concentration is doubled to 0.27 mA/cm^2^ at 1.23 VRHE. However, the photocurrents of the other two samples are small. Their photocurrents reach at 0.10 mA/cm^2^ (ZnO(C)-2A) and 0.09 mA/cm^2^ (ZnO(C)-3A), respectively. Although the photoelectric properties of the three electrodes can be improved by reducing their oxygen vacancies, the photocurrent of the four samples (ZnO(C)-2 (3, 2A or 3A)) is still much lower than that of the two samples ZnO(C)-1 (or 1A). According to the previous test results, when the sintering temperature of the ceramic reaches 900 °C, the zinc element in ZnO will decompose and volatilize, and become interstitial zinc (Zni) defects, which improves the conductivity of the ceramic, and the color of the ceramic will also become dark yellow, but the increase of the type of defects and the concentration of oxygen defects destroy the integrity of the crystal structure of ZnO. At 1100 °C, the concentration of interstitial zinc (Zni) is greatly increased, which is the main defect, and the color of ceramic becomes reddish brown. Even if the sample (ZnO(C)-3) is heated in the air, its color does not change. In addition, among the six ZnO ceramic electrodes, only ZnO(C)-1 and ZnO(C)-1A samples have a photoelectric response to visible light; however, the two photocurrents are very small (Figure S6B and Table S4), and the ratio Iv/I1.5 is less than 4%, which also decreases with the decrease in oxygen defect concentration. This indicates that the photoelectric response of the six samples is mainly caused by the intrinsic light absorption of ZnO. This is consistent with that of Lu et al. [14]. Of course, the appropriate concentration of oxygen vacancy has a certain contribution to the photoelectric properties of ZnO. This work will help researchers to deepen the research on the control and formation of defects in ZnO, and provide necessary reference for the research of photoelectric properties of ZnO materials.

## 4. Conclusions

In summary, the defect-adjustable ZnO ceramics were prepared by the SPS method. By changing the sintering temperature and using the reduction environment, different concentrations of defects are introduced into the ceramic. The higher the defect concentration in ZnO ceramics, especially the interstitial zinc defects, the higher the electrical conductivity. The photoelectric characterization shows that the higher the defect concentration, the worse the photoelectric performance. The photocurrent of its photoelectric response mainly comes from the intrinsic light absorption of ZnO. Moreover, the photocurrent of visible light only exists in the presence of appropriate concentration of oxygen vacancies, and the contribution of photocurrent is very small. The work studied the defects and the electrical and photoelectric properties of ZnO ceramics, which will provide useful reference for researchers to study the different properties of zinc oxide materials. With the tunable visible light response, it is reasonably believed that ZnO will find great application potential in the fields ranging from energy storage to heterogeneous catalysis and photoelectrochemistry.

## Data Availability

The data presented in this study are available on request from the corresponding author.

## Supplementary Materials

The following are available online at https://www.mdpi.com/article/10.3390/nano11102506/s1, Figure S1: XRD patterns of the ZnO(C)-2A (3A) samples, ZnO(C)-1B (2B and 3B) samples, ZnO(C)^1^, ZnO(C)^2^ and ZnO(C)^3^ and the deposits on the inner surface of quartz sheet, Table S1: The quality changes of the three samples after 1h treatment in 1100 °C vacuum condition, Figure S2: UV-Vis DRS of the ZnO(C)-2A (3A) samples, the ZnO(C)-1B (2B and 3B) samples and the ZnO(C)^1^, ZnO(C)^2^ and ZnO(C)^3^ samples, Figure S3: SEM images of the ZnO(C)-1 (2 and 3) samples and the ZnO(C)-1B (2B and 3B) samples, Table S2: The density (ρ) and relative density of the ZnO powder and ceramics prepared at 700 °C, 900 °C and 1100 °C, Figure S4: XPS patterns of the ZnO powder and ZnO(C)-1A (2A and 3A) and the ZnO(C)-1B (2B and 3B), Figure S5: (A) Raman spectrum of ZnO(C)-1A (2A and 3A) samples, ZnO(C)-1B (2B and 3B) samples and ZnO(C)^1^, ZnO(C)^2^ and ZnO(C)^3^ samples, Table S3: The resistivity mobility and density results of ZnO(C)-1, ZnO(C)-2 and ZnO(C)-3, Figure S6: Photocurrent density versus applied potential (J-V) curves measured for the ZnO(C)-1A (2A and 3A) and Photocurrent density versus applied potential (J-V) curves measured for the ZnO(C)-1 (2 and 3) and ZnO(C)-1A (2A and 3A) photoanodes under visible light, Table S4: Photocurrents (I1.5 and Iv) of ZnO(C)-1(1A) under the AM 1.5G illumination and visible light at 1.23 VRHE.
