# Supplementary material for: Fabrication of ZnO Ceramics with Defects by Spark Plasma Sintering Method and Investigations of Their Photoelectrochemical Properties
Source: Nanomaterials (Basel). 2021 Sep 26;11(10):2506. doi: 10.3390/nano11102506 (PMC8537134; doi:10.3390/nano11102506)
Supplement: Supplementary file 1 [file nanomaterials-11-02506-s001.zip › nanomaterials-1375196-supplementary.pdf]

nanomaterials

Supplementary Materials

Fabrication of ZnO Ceramics with Defects by Spark Plasma Sintering Method and Investigations of Their Photoelectrochemical Properties

Liren Zheng [1,2], Mu Liu [1], Haipeng Zhang [1], Zhaoke Zheng [1], Zeyan Wang [1,*], Hefeng Cheng [1], Peng Wang [1], Yuanyuan Liu [1] and Baibiao Huang [1,*]

1 State Key Laboratory of Crystal Materials, Shandong University, Jinan 250100, China; 201720279@mail.sdu.edu.cn (L.Z.); liumu@mail.sdu.edu.cn (M.L.); 201820321@mail.sdu.edu.cn (H.Z.); zkzheng@sdu.edu.cn (Z.Z.); chenghefeng@sdu.edu.cn (H.C.); pengwangicm@sdu.edu.cn (P.W.); yyliu@sdu.edu.cn (Y.L.)
2 School of Physics and Electronic Engineering, Taishan University, Tai'an 271000, China
* Correspondence Email: wangzeyan@sdu.edu.cn (Z.W.); bbhuang@sdu.edu.cn (B.H.)

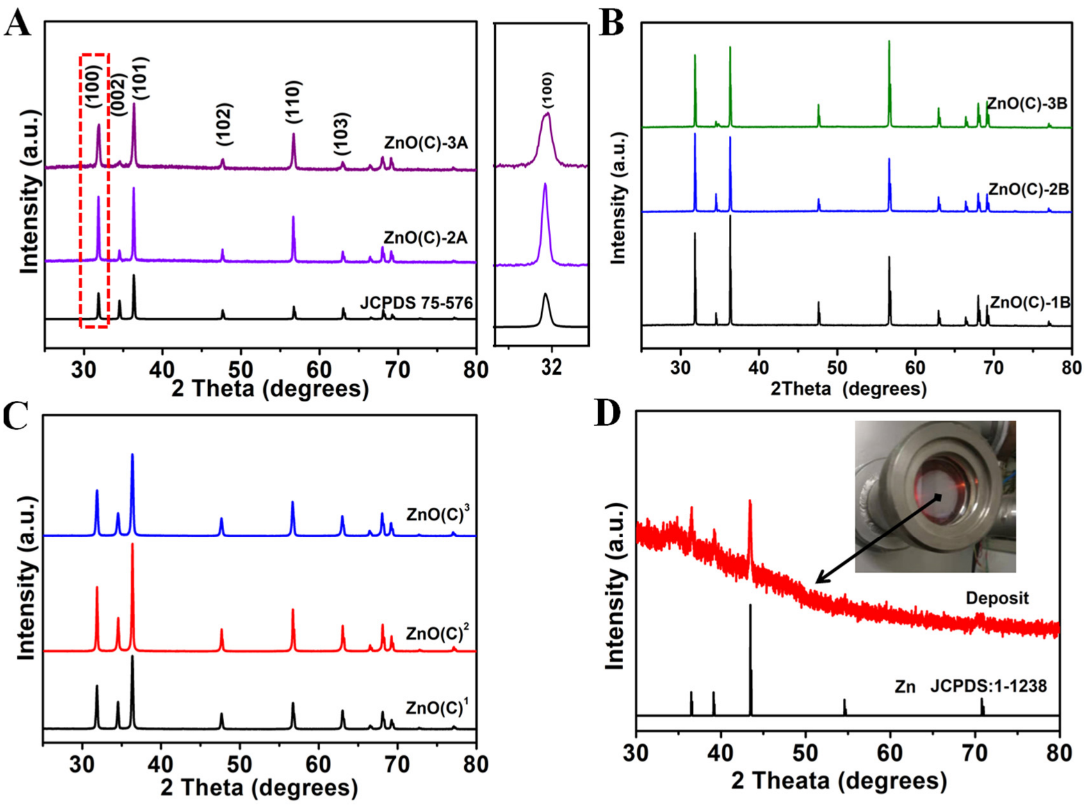

Figure S1. (**A**) XRD patterns of the ZnO(C)-2A (3A) samples; (**B**) XRD patterns of the ZnO(C)-1B (2B and 3B) samples; (**C**) XRD patterns of the ZnO(C)[1], ZnO(C)[2] and ZnO(C)[3] samples; (**D**) XRD of the deposits on the inner surface of quartz sheet. insert: digital photograph of the observation window of a vacuum furnace with silver-gray zinc deposited on the inner surface of the quartz glass.

Table S1. The quality changes of the three samples after 1h treatment in 1100 °C vacuum condition.

	ZnO(C)-1	ZnO(C)-2	ZnO(C)-3
before	39.2 mg	44.7 mg	45.5 mg
after	28.5 mg	35.3 mg	36.1 mg

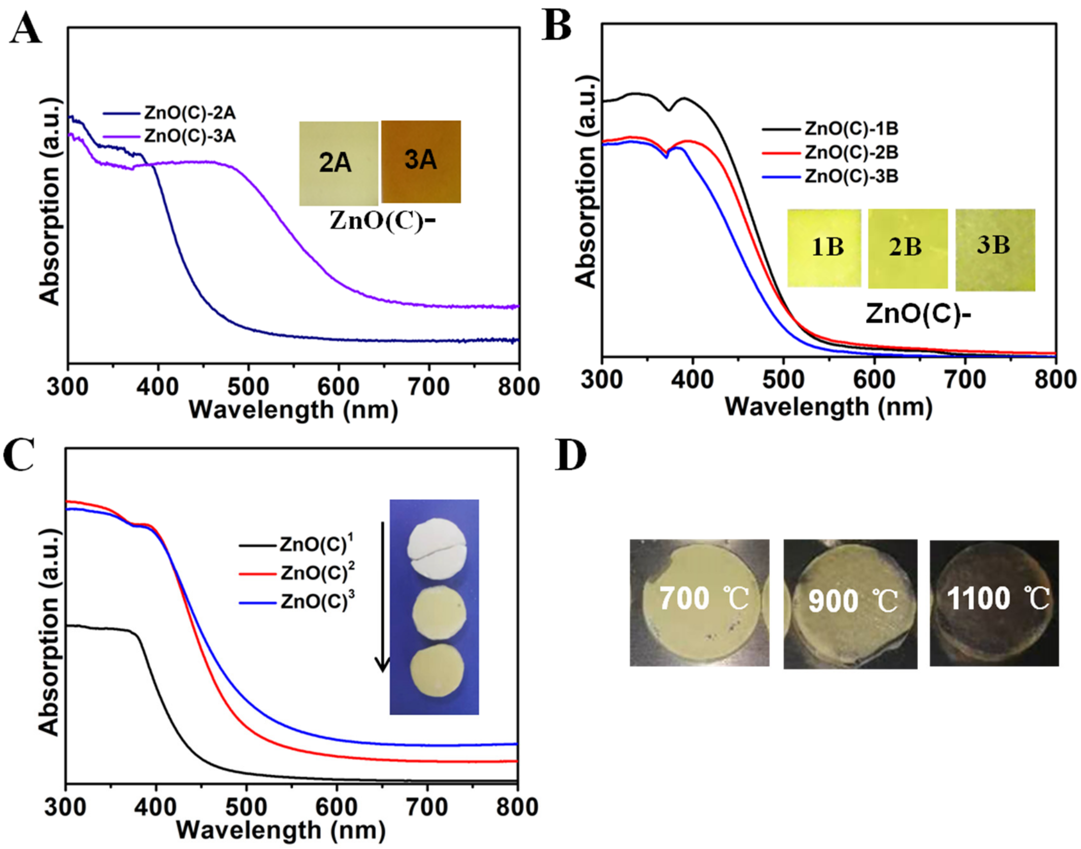

Figure S2. (**A**) UV-Vis DRS of the ZnO(C)-2A (3A) samples, insert: the digital photographs of ZnO(C)-2A (3A); (**B**) UV-Vis DRS of the ZnO(C)-1B (2B and 3B) samples, insert: the digital photographs of ZnO(C)-1B (2B and 3B); (**C**) UV-Vis DRS of the ZnO(C)^{1}, ZnO(C)^{2} and ZnO(C)^{3} samples, insert: the digital photographs of the three samples; (**D**) The digital photographs of three ceramics prepared at different temperatures, with graphite paper around them cleaned with sandpaper.

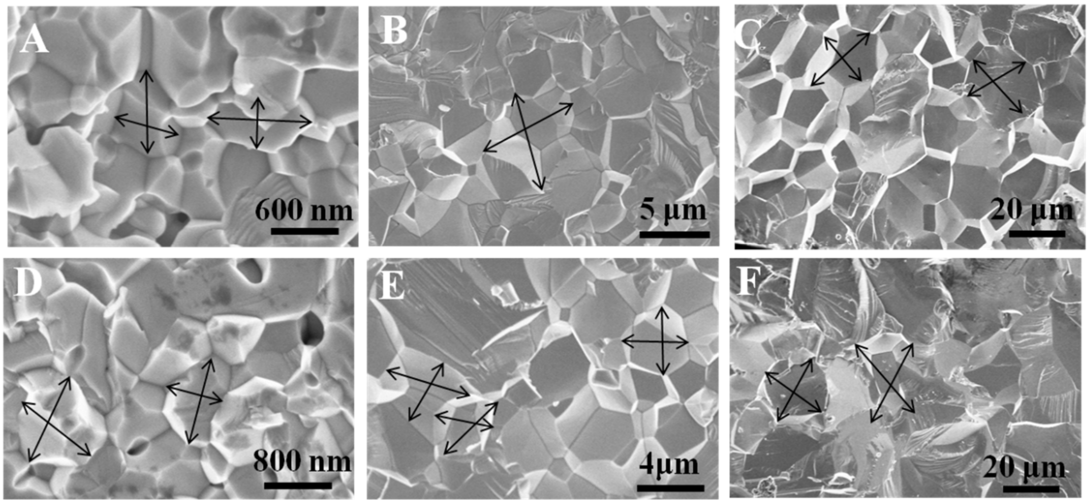

Figure S3. SEM images of the ceramics obtained at deferent temperature and the ceramics annealed at 500 °C for 5 h in ambient, (**A**) ZnO(C)-1, (**B**) ZnO(C)-2, (**C**) ZnO(C)-3, (**D**) ZnO(C)-1A, (**E**) ZnO(C)-2A and (**F**) ZnO(C)-3A. Insert: the " ⟷ " represents the auxiliary line for grain size measurement.

Table S2. The density (ϱ) and relative density of the ZnO powder and ceramics prepared at 700 °C, 900 °C and 1100 °C.

Temperature	700 °C		900 °C		1100 °C	
Density and Relative density	ϱ (g/cm³)	Relative density (%)	ϱ (g/cm³)	Relative density (%)	ϱ (g/cm³)	Relative density (%)
SPS method	5.54	98.1	5.65	99.6	5.70	100
Traditional sintering method	4.90	86.7	5.60	99.1	5.65	99.6

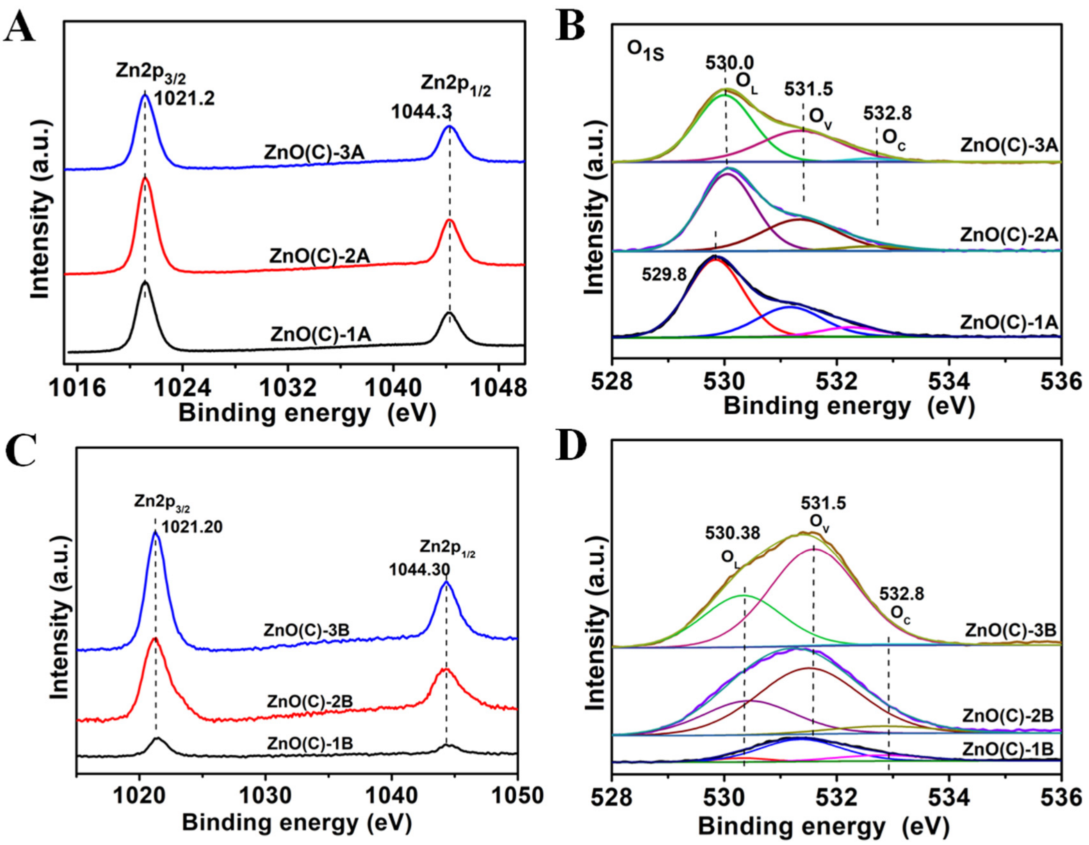

Figure S4. XPS patterns of the ZnO powder and ZnO(C)-1A (2A and 3A), (**A**) Zn 2p peak and (**B**) O1s peak; XPS patterns of the ZnO(C)-1B (2B and 3B), (**C**) Zn 2p peak and (**D**) O1s peak.

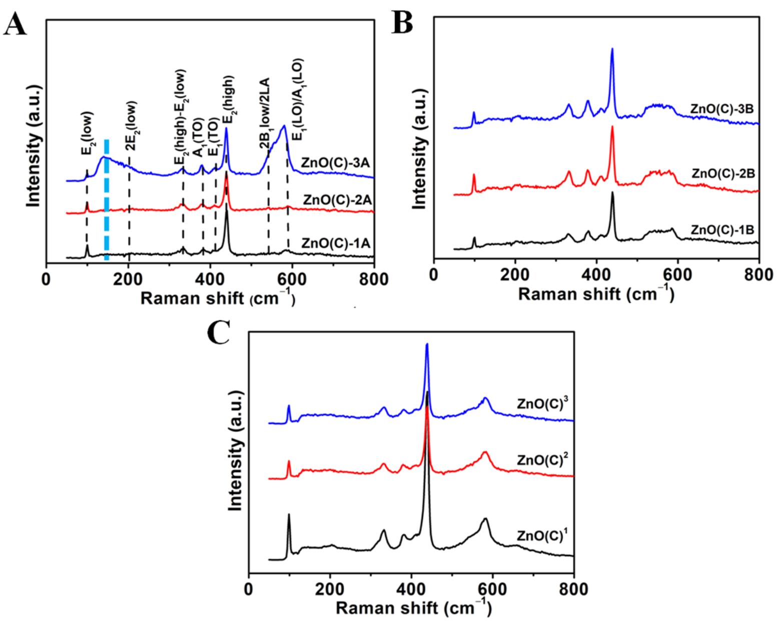

Figure S5. (**A**) Raman spectrum of ZnO(C)-1A (2A and 3A) samples; (**B**) Raman spectrum of ZnO(C)-1B (2B and 3B) samples; (**C**) Raman spectrum of ZnO(C)[1], ZnO(C)[2] and ZnO(C)[3] samples.

Table S3. The resistivity mobility and density results of ZnO(C)-1, ZnO(C)-2 and ZnO(C)-3.

	ZnO(C)-1	ZnO(C)-2	ZnO(C)-3
**Resistivity (Ω·cm)**	1187.52	0.1435	0.0561
**Mobility (cm^2/vs)**	7.94	26.63	38.94
**Density (cm^{-3})**	6.65×10^{14}	1.63×10^{18}	2.86×10^{18}

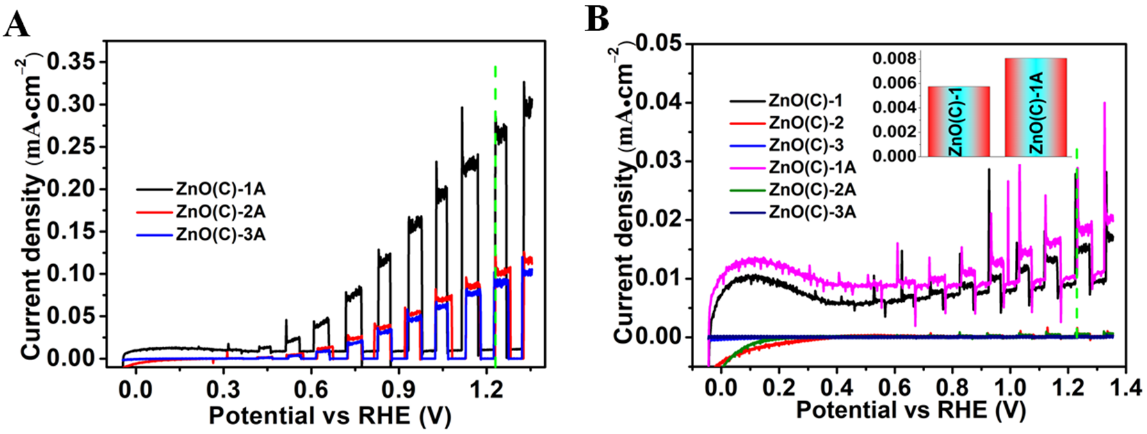

Figure S6. (**A**) Photocurrent density versus applied potential (J-V) curves measured for the ZnO(C)-1A (2A and 3A) photoanodes with a scan rate of 20 mV/s under AM 1.5G illumination or visible light irradiation in 0.1 M $NaSO_4$ solution(PH = 6.8) and (**B**) Photocurrent density versus applied potential (J-V) curves measured for the ZnO(C)-1 (2 and 3) and ZnO(C)-1A (2A and 3A) photoanodes with a scan rate of 20 mV/s under visible light irradiation by 300W Xe arc lamp equipped with a UV/IR cut filter (λ≥ 420 nm) inset, the photocurrent values at 1.23 V_{RHE} .

Table S4. Photocurrents ($I_{1.5}$ and I_v) of ZnO(C)-1(1A) under the AM 1.5G illumination and visible light at 1.23 V_{RHE}.

	$I_{1.5}$ (mA/cm^2)	I_v (mA/cm^2)	$I_v/I_{1.5}$
**ZnO(C)-1**	0.14	0.005	3.6 %
**ZnO(C)-1A**	0.27	0.008	2.9 %
